# Peritoneal Dialysis Catheter-Associated Peritonitis Caused by Mycobacterium abscessus: A True Infection?

**DOI:** 10.7759/cureus.68721

**Published:** 2024-09-05

**Authors:** Ei Khin, Rosa Rodriguez, Sarah K Walker, Gilbert Handal

**Affiliations:** 1 Pediatric Nephrology, Texas Tech University Health Sciences Center El Paso Paul L. Foster School of Medicine, El Paso, USA; 2 Pediatric Nephrology, El Paso Children's Hospital, El Paso, USA; 3 Pediatrics, Texas Tech University Health Sciences Center El Paso Paul L. Foster School of Medicine, El Paso, USA; 4 Surgery, Texas Tech University Health Sciences Center El Paso Paul L. Foster School of Medicine, El Paso, USA

**Keywords:** atypical mycobacterium, mycobacterium abscessus complex, mycobacterium species, non-tuberculous peritonitis, peritoneal dialysis catheter associated peritonitis

## Abstract

A nine-year-old male with trisomy 21, end-stage renal disease (ESRD) due to reflux nephropathy presented with suspected peritoneal dialysis (PD) catheter-associated peritonitis. One week after receiving an intraperitoneal antibiotic, he presented again with persistent peritonitis symptoms and bloody PD fluid. He underwent exploratory laparotomy, abdominal washout, and PD catheter removal. *Mycobacterium abscessus *(*M. abscessus)* was found in the intraoperative peritoneal fluid culture. After the catheter removal, the child’s symptoms significantly improved without antimicrobial medications. He was maintained on hemodialysis three times a week and underwent a successful kidney transplant eight months after this episode. Non-tuberculous mycobacterial peritonitis should be considered in patients with culture-negative peritonitis when there is no intraperitoneal antibiotic response. *M. abscessus* is a rapidly growing atypical *Mycobacterium* found in the environment and can contaminate medical devices. Our case involved an infection from a contaminated PD catheter since the patient's symptoms improved after PD catheter removal.

## Introduction

Peritoneal dialysis (PD) is a treatment modality for kidney failure, where the peritoneum acts as a filter to remove waste from the blood. Dialysis fluid is introduced into the abdominal cavity through the catheter, which absorbs waste and is then drained out. PD catheter-associated peritonitis can be a serious complication for patients undergoing PD. It can cause catheter loss, peritoneal scarring, PD failure, high hospitalization costs, premature transition to hemodialysis, and even death. In one adult study, peritonitis was associated with a 95% increased risk of all-cause mortality in patients with PD [1].

Coagulase-negative staphylococci, *Staphylococcus aureus*, and *Pseudomonas aeruginosa* are the most commonly associated pathogens. Culture-negative peritonitis accounts for 0-30% of cases [2]. Of those infections with positive cultures, mycobacteria either tuberculous or atypical [non-tuberculous mycobacteria (NTM)], account for less than 3%. Non-tuberculous mycobacteria refer to mycobacterial species other than *Mycobacterium tuberculosis* and *Mycobacterium leprae* [3]. These are ubiquitous environmental organisms found in soil, water, dust, medical equipment, and surgical solutions. Suresh et al. have reported that *Mycobacterium abscessus* (*M. abscessus)* (33.3%) was the most commonly isolated of the 19 NTM species [4].

The spectrum of clinical presentations, treatment options, and patient outcomes can vary depending on which NTM species is involved. *M. abscessus *can cause various infections in several organs. It can be resistant and refractory to treatment with antimicrobial drugs. The duration of therapy remains unclear for this infection and PD catheter removal is crucial [5,6]. Some case reports have described *M. abscessus* PD catheter-associated peritonitis managed with prolonged antimicrobial treatment and PD catheter removal. We present a case of PD-associated catheter infection caused by *M. abscessus* that was successfully treated with catheter removal and abdominal washout.

## Case presentation

A nine-year-old male with trisomy 21, end-stage renal disease (ESRD) due to reflux nephropathy, presented with fever, fatigue, and decreased oral intake for two days. He had been on PD for 10 months and had experienced *Staphylococcus aureus* PD-associated peritonitis four months before this episode. He had undergone the removal of a portion of the PD catheter cuff due to partial cuff erosion three months before this presentation. At this presentation, he was seen by a pediatrician to evaluate a fever and was prescribed amoxicillin. A sample of PD fluid was obtained on the second day of fever at an outpatient dialysis center. (Of note, the PD fluid was not cloudy.)

We performed PD fluid cell count and culture. The white blood cell (WBC) count of PD fluid was 243 with a predominance of higher monocyte count (86%) and 14% segmented cells. The culture did not grow any organism. After the sample was taken, outpatient intraperitoneal (IP) ceftazidime and cefazolin were started. Due to the persistent fever despite IP antibiotic treatment, the patient was admitted to the hospital on day three to rule out sepsis. Complete blood count showed no leukocytosis or bands. the inflammatory markers were as follows: C-reactive protein (CRP): 6.30 mg/dL (normal range: 0-1) and erythrocyte sedimentation rate (ESR): 85 (normal level: <10 mm/hr). The urinalysis was normal and urine culture and blood culture remained negative. The abdominal ultrasound was unremarkable. A follow-up PD culture was negative, and the patient was kept on IP ceftazidime and cefazolin.

One week later, the patient was readmitted with persistent high fever, generalized abdominal pain, scrotal pain, newly bloody PD fluid, and diarrhea for three days. On examination, the patient had abdominal tenderness and enlargement. The PD fluid showed nucleated cells of 1756; 76% of body fluid was segmented cells and 24% of body fluid was monocytes. There was no redness, discharge from the exit site, or tenderness along the catheter tunnel. The cultures of the PD fluid including fungal were negative, and therapy was switched to IP vancomycin and gentamicin.

**Figure 1:**
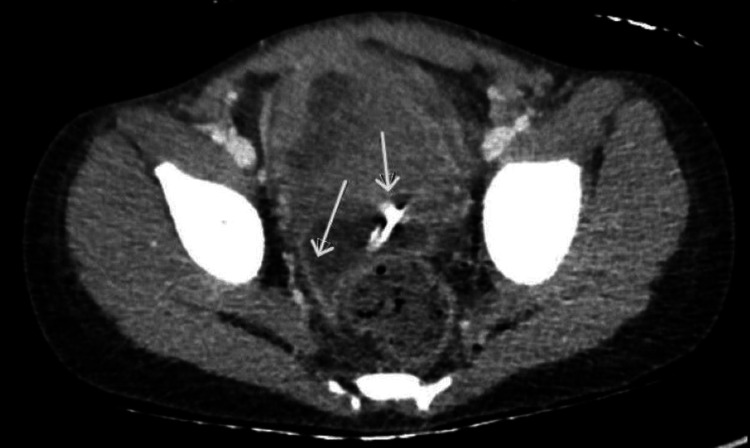
CT abdomen and pelvis (right arrow) showing PD catheter with enhancement of the nearby peritoneal wall (left arrow) CT: computed tomography; PD: peritoneal dialysis

**Figure 2:**
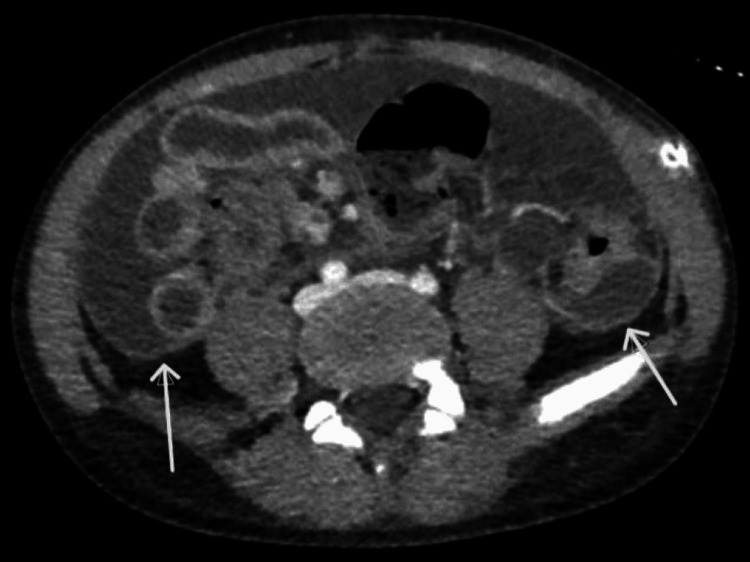
CT abdomen and pelvis (arrows) showing peritoneal wall enhancement with ascites CT: computed tomography

Due to bloody PD fluid, a pediatric surgeon was consulted. CT of the abdomen and pelvis was done to rule out catheter erosion causing visceral perforation or bleeding. CT findings (Figure 1) showed PD catheter and enhancement of the nearby peritoneal wall. As shown in Figure 2, we observed peritoneal wall enhancement and ascites consistent with acute peritoneal inflammatory/infectious process and diffuse enteritis. Due to concerns that the catheter may have eroded into a structure or vessels, exploratory laparoscopy was scheduled, along with abdominal washout, peritoneal dialysis catheter removal, and hemodialysis catheter placement. The patient received intravenous meropenem, vancomycin, and fluconazole during the second admission. An intraoperative peritoneal fluid culture demonstrated acid-fast bacilli, which was positive for *Mycobacterium abscessus.* PD fluid cell count and culture report are shown in Table 1.

**Table 1:** Peritoneal dialysis fluid cell count and culture Normal peritoneal fluid is a transparent straw-colored ultrafiltrate of plasma. According to the International Society of Peritoneal Dialysis (ISPD) guidelines, dialysis effluent white cell count >100/µL with >50% polymorphonuclear leukocytes (PMN)/segmented body fluid % is significant for peritonitis PD: peritoneal dialysis

Peritoneal fluid (PD)	Initial diagnosis	Second hospital admission (one week after initial diagnosis)
Color	Yellow	Yellow
Appearance	Clear	Cloudy
Total nucleated cells	243	1756
RBCs body fluid (BF)	0	78
Segmented BF %	14	76
Monocyte BF %	86	24
PD fluid culture	Negative	Negative
Blood culture	Negative	Negative
Fungal culture	Negative	Negative
Mycobacterium culture (from intraoperative peritoneal fluid)	Not done	Mycobacterium abscessus

Infectious disease (ID) was consulted regarding *M. abscessus* finding. After catheter removal, the patient's abdominal pain, diarrhea, and fever resolved. He was not treated for the NTM infection with combined antimicrobial treatment. We followed up on clinical symptoms such as fever, abdominal pain, and weekly blood culture while he was on hemodialysis. ID specialists followed mycobacteria nuclear antigen tests and collected peritoneal fluid culture follow-up for three months to rule out slowly growing NTM organisms. No additional organism was detected. Since he had been evaluated for a kidney transplant, the pediatric nephrologist and the patient's family decided to continue with hemodialysis. The patient received a kidney transplant eight months after this episode of infection. He is now post-kidney transplant for three years and has been doing well despite immunosuppression.

## Discussion

According to the International Society of Peritoneal Dialysis (ISPD) guidelines, peritonitis should be diagnosed when at least two of the following are present: 1. clinical features consistent with peritonitis, that is, abdominal pain and/or cloudy dialysis effluent; 2. dialysis-effluent white cell count >100/µL or > 0.1 × 10^9^/L (after a dwell time of at least two hours), with >50% polymorphonuclear leukocytes (PMN); 3. positive dialysis-effluent culture [7]. The majority of infections associated with peritoneal dialysis are Gram-positive or negative bacteria. About 10-20% of the cultures detect no pathogenic bacteria, and in such cases, it is advisable to evaluate for fastidious organisms such as atypical mycobacteria, *Nocardia*, or filamentous fungi. Exit site infection has been defined as the presence of purulent discharge with or without erythema of the skin at the catheter-epidermal interface [8]. In our patient, no signs such as purulent discharge, tenderness, erythema, swelling, or induration around the catheter tract were appreciated. He persistently had symptoms of peritonitis despite receiving treatment with IP antibiotics and a negative bacterial culture from a PD fluid sample. Additionally, there is a high prevalence of tuberculosis in our geographical area, and PD fluids were sent for acid-fast stain.

*M. abscessus* is a rapidly growing NTM and takes only three to seven days to grow. It is frequently found in the environment, dust, water, dialysate fluid, surgical solution, and soil [9,10]. The most common clinical presentations are skin, soft tissue, and respiratory infections [4]. We continued to follow up on the AFB culture media for three months to rule out slowly growing organisms as well. *M. abscessus* infection can be resistant, and refractory to combined antimicrobial treatment. Its treatment duration is usually long and multidrug therapy is required for its eradication. The treatment previously described for NTM infection includes macrolides, tetracyclines, aminoglycosides, fluoroquinolones, cefoxitin, imipenem, rifampin, ethambutol, and rifabutin [2]. The use of such drugs can pose a management challenge as some patients are already suffering from renal failure and are on dialysis.

After the removal of the catheter, our patient had no further symptoms of peritoneal irritation or fluid accumulation. Distinguishing between catheter infection and peritonitis is essential as the prognosis and treatment of the two conditions can be different. The treatment of peritonitis should be aggressive, requiring at least triple antibiotic therapy for eight weeks or more at the minimum until the infection is eradicated. We ultimately concluded that our case was limited to PD catheter infection without full-blown peritonitis. The decision whether or not to treat must be made with caution, particularly in patients who need a transplant, as an active atypical mycobacteria infection would be a contraindication for a kidney transplant. In our case, we decided not to provide treatment with combined antimicrobial medications.

We observed the patient cautiously with clinical examination, weekly blood cultures, and mycobacterial nuclear antigen testing. There was no fluid collection on the abdominal ultrasound a week after the catheter removal. The collected PD fluid mycobacterial culture was monitored for any additional organism’s growth for three months. The patient continued to be on hemodialysis and underwent follow-up blood cultures weekly. PD was not restarted and he received a kidney transplant eight months after this infectious episode.

## Conclusions

The diagnosis of non-tuberculous mycobacterial peritonitis can be challenging as it has no specific signs or symptoms. However, a high index of suspicion is required in patients with culture-negative peritonitis who are unresponsive to empirical antibiotics. Our patient was treated successfully with PD catheter removal and abdominal washout.
